# Application of fibrin-based hydrogels for nerve protection and regeneration after spinal cord injury

**DOI:** 10.1186/s13036-020-00244-3

**Published:** 2020-08-03

**Authors:** Ziyuan Yu, Hongru Li, Peng Xia, Weijian Kong, Yuxin Chang, Chuan Fu, Kai Wang, Xiaoyu Yang, Zhiping Qi

**Affiliations:** Department of Orthopedic Surgery, The Second Hospital of Jilin University, Ziqiang Street No. 218, Changchun, TX 130041 PR China

**Keywords:** Fibrin hydrogels, Spinal cord injury, Topographical guidance, Stem cells delivery, Cytokines delivery, Drug delivery

## Abstract

Traffic accidents, falls, and many other events may cause traumatic spinal cord injuries (SCIs), resulting in nerve cells and extracellular matrix loss in the spinal cord, along with blood loss, inflammation, oxidative stress (OS), and others. The continuous development of neural tissue engineering has attracted increasing attention on the application of fibrin hydrogels in repairing SCIs. Except for excellent biocompatibility, flexibility, and plasticity, fibrin, a component of extracellular matrix (ECM), can be equipped with cells, ECM protein, and various growth factors to promote damage repair. This review will focus on the advantages and disadvantages of fibrin hydrogels from different sources, as well as the various modifications for internal topographical guidance during the polymerization. From the perspective of further improvement of cell function before and after the delivery of stem cell, cytokine, and drug, this review will also evaluate the application of fibrin hydrogels as a carrier to the therapy of nerve repair and regeneration, to mirror the recent development tendency and challenge.

## Introduction

The spinal cord is an integral part of the central nervous system (CNS) regulating sensory and motor function. Worldwide, 760,000 new cases of traumatic SCI occur each year as a result of traffic accidents, falls and other causes [1]. Traumatic and non-traumatic SCIs often result in limb paralysis below the injured neurological segmental level, incontinence, and sexual dysfunction [2]. Sensory function or motor function loss seriously affects the patients’ life quality. The medical treatments, lifelong nursing, rehabilitation training, and other consequences put a large cost on family and society [3]. Clinical treatments of early decompressive surgery, ictus treatment of large dose methylprednisolone, exercise training, epidural stimulation, and others are to prevent the secondary death of remaining nerves or to enhance the compensation of spared circuits function [4–7]. Studies on SCIs have exhibited extreme limitation on the axon regeneration in the injured adult mammalian CNS [8]; The process of injured axons regeneration is similar to the process of axon growth during CNS development. As the size of the spinal cord increases in adulthood, axons regeneration often takes a long time to extend longer distances [9]. Besides, the surrounding inhibitory environment prevents the regenerating axon from re-connecting with the target tissue [10]. Therefore, achieving new functional connections and eliminating the inhibition from the surrounding environment have turned out a significant challenge for regenerative medicine [11].

In neural tissue engineering, multifunctional scaffolds capable of carrying cells and therapeutic molecules serve as an ideal solution to the above problems. Synthetic polymer scaffolds with fewer impurities and batch differences, controllable flexibility, and mechanical strength, have achieved extensive application in nerve repair [12, 13]. However, shortcomings of incomplete polymerization, harmful degradation products, and toxic residues caused by plasticizers exist in synthetic polymer scaffolds [14, 15]. Fibrin tissue sealant is a member of natural polymer and ECM, and the US Food and Drug Administration (FDA) approved it for intraoperative hemostasis and wound repair in 1998 [16]. Except for high biocompatibility, the binding sites of many cells, ECM proteins, and growth factors make fibrin hydrogels modifiable [17]. The tunable ratio of fibrinogen to thrombin can adjust fibrin hydrogel’s mechanical properties to match human spinal cord tissue’s mechanical properties. The elastic modulus of the human spinal cord is about 40.12 ± 6.90 kPa, and the maximum/failure stress is about 62.26 ± 5.02 kPa [18]. Fibrin hydrogels can shape up in vitro or inside the spinal cord after injection. The shape of in-situ polymerized scaffolds can conform to the defect tissue to create an integrative implant-tissue interface [19].

**Fig. 1:**
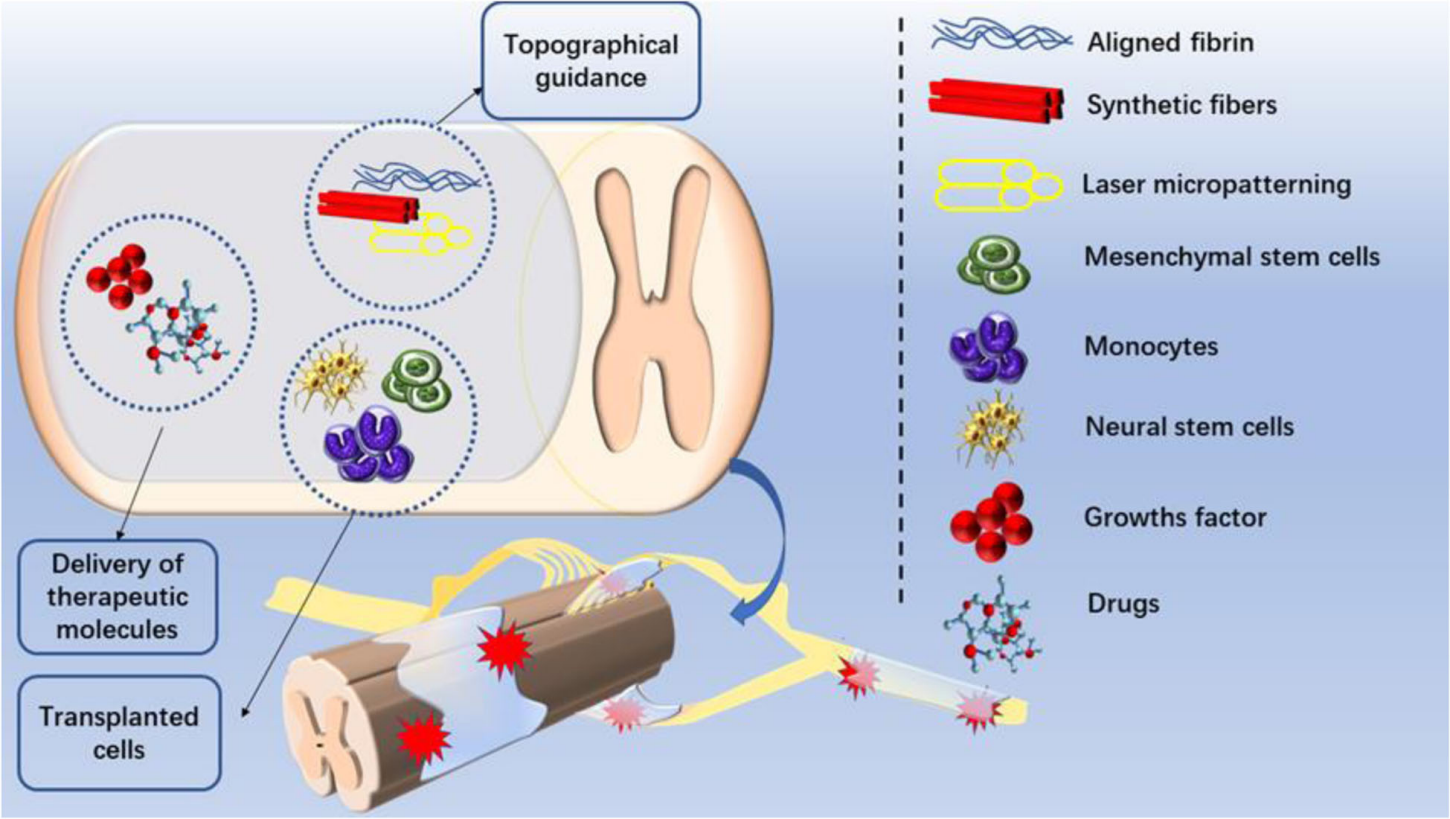
The application of fibrin-based hydrogel in nerve protection and regeneration. Fibrin-based hydrogels can promote the repair of spinal cord injury and peripheral nerve injury. Researchers have developed different methods to improve the repair capacity of fibrin hydrogels, including transplanting cells, carrying therapeutic molecules, and giving hydrogel internal topographical guidance

Moreover, the by-products released by fibrin hydrogels during the polymerization or in vivo degradation can facilitate the repair of damage [17]. However, the rapid degradation rate and weak mechanical properties of fibrin hydrogels make it unsuitable for application in bone tissue engineering and cartilage tissue engineering alone. Researchers usually combine fibrin hydrogels with stable and hard materials to overcome the limitations [20]. Nevertheless, fibrin has great modification potential in terms of physical and chemical properties. This report reviews the application of hydrogels composed of fibrin as the primary component in nerve repair. We have also summarized the advantages and disadvantages of various modification methods to provide a reference for better future development (Fig. 1).

## Physiological changes after spinal cord injury

Pathology divides traumatic SCI into initial trauma and secondary injury. Spinal cord compression or transection caused by mechanical damage from initial trauma leads to cell death in the spinal cord and blood-spinal cord barrier dysfunction. The occurrence of a secondary injury cascade following the initial trauma is characterized by ischemia, oxidative stress, inflammatory cell infiltration, and molecule release and impaired ionic homeostasis [2, 11]. In the acute phase of spinal cord injury, astrocytes proliferate and infiltrate in the lesion site to participate in the formation of the glial scars, the inhibitory factors expression, interstitial cell apoptosis and necrosis, excitotoxicity. Moreover, the interruption of axoplasmic transport causes the accumulation of internal calcium ions to trigger Wallerian degeneration, which makes axon retract from the lesion site, resulting in cystic cavitation [21]. The physical inhibition of the surrounding environment caused by the glial scars, the chemical inhibition caused by chondroitin sulfate protein (CSPG), and myelin-associated inhibitors (MAIs), are identified as the primary environmental factors impeding nerve regeneration and circuit reconstruction [22, 23].

The glial scar consists of activated glial cells, meningeal cells, pericytes, and macrophages, creating a physical barrier for axon regeneration [24–26]. The glial scar is rich in CSPG, inhibiting axons and myelin sheaths growth, and oligodendroglia cell maturation [27]. Differing from the hard scars caused by other tissue injuries, spinal cord scars exhibit softer mechanical characteristics than the original tissues. Studies suggest that the soft matrix makes stem cells more likely to differentiate into glial cells rather than neurons [28]. Surgical removal of the glial scars can reduce the inhibition to some extent [29]. However, studies illustrated the protective effects of glial scars on CNS [26, 30]. Glial scars can protect the normal nerve tissue, inhibit the inflammation damage, and facilitate the repair. Sole inhibition on glial scar formation may lead to microenvironment deterioration.

MAIs such as Nogo-A, oligodendrocyte-myelin glycoprotein, myelin-associated glycoprotein, and others are repulsive guidance cues in central nervous system maturation. MAIs can not only guide and regulate the correct migration of neurons and appropriate myelination but also stabilize the functional neuronal circuits. After SCI, the up-regulation of MAIs in neurons and oligodendrocytes at the injury site inhibits the myelination and the nerve re-innervation [31, 32].

## Application requirements of hydrogels in tissue engineering

Hydrogels are cross-linked by hydrophilic polymer chains in an aqueous microenvironment, with porosity and excellent water retention [33]. The easy modification of physicochemical property, the high capacity to imitate the natural ECM, and the excellent polymerization in situ at the injury site to acquire a structure better matching the stump have moved hydrogel in favor of the application to tissue engineering [34, 35]. Compared with the repair methods for single aplasia, the versatility of hydrogel can promote the repair of damage in many approaches. Hydrogels composed of fibrin and other natural polymers can serve as scaffolds in tissue engineering Table 1, Table 2.

**Table 1:** Fibrin combined with natural polymers as a scaffold in tissue engineering

Composite material	Fabrication methodology	Architecture	Enhanced performance	Achievements	References
Alginate	3D bioprinting	Hollow gel tube-like structure, the inner layer of alginate, and the outer layer of fibrin	Long-term cell viability	Construction of artificial arteries and veins	[36]
			Biocompatibility		
			Cell adhesion		
Chitosan	Mix	Fibrin hydrogels embedded with Sonic hedgehog (SHH)-loaded chitosan	Delay the release of SHH,	Promote spinal cord regeneration	[37]
			Nerve regeneration		
			Recovery of motor function,		
			Reduce tissue cavities		
Collagen	Fill conduits with fibrin hydrogels	Collagen conduits filled with fibrin hydrogels	The intensity of scaffold,	Nerve conduits for peripheral nerve regeneration	[38]
		Culture autologous adipose-derived mesenchymal stem cells (ADMSCs)	Expression of protein GAP-43		
			Axon regeneration		
Fibronectin	Mix	Injectable forms of fibrin and fibronectin hydrogels	Integrate with damaged spinal cord tissue,	Injectable materials to fill cavities in the spinal cord	[39]
			Axon growth		
Gelatin	Mix	Bone matrix gelatin mixed fibrin acts as a cell culture substrate	Biocompatibility	As a scaffold in cartilage tissue engineering	[40]
			Production of collagen II and aggrecan		
Hyaluronic acid	3D bioprinting	Core-shell structure, Neuronal cells and Schwann cells are respectively located in the core and shell	Neurogenesis,	Biomimetic nerve fibers with a bionic tubular myelin sheath	[41]
			Myelin maturation,		
			Coexistence of Schwann cells and neurons		
Albumin	Mix	Albumin fibrin hydrogels embedded in the ferromagnetic fiber network	Extracellular matrix deposition	Scaffold for bone tissue engineering	[42]
			Vascularisation

**Table 2:** Fibrin combined with synthetic polymers as a scaffold in tissue engineering

Composite material	Fabrication methodology	Architecture	Enhanced performance	Achievements	References
Poly (DL-lactic-co-glycolic acid) (PLGA)	Electrospray for PLGA microspheres,	Aligned fibrin hydrogels loaded with PLGA microspheres,	Reduce the initial burst release of drugs	Promote spinal cord regeneration	[43]
	Electrospinning for aligned fibrin hydrogels		Stem cells extend along the long axis of the aligned hydrogels		
		PLGA microspheres contain drugs			
		Culture stem cells			
Poly(L-lactide) (PLLA)	Vacuum deposition	Fibrin deposits on the microporous walls of PLLA scaffolds	Increase elastic modulus	Promote the early regeneration of bone and cartilage tissue	[44]
			Cell adhesion		
Polylactic Acid (PLA)	Melt-spun for PLA fibers	Fibrin hydrogels contain square PLA fibers	Structural support	Biomaterials for cardiovascular tissue engineering	[45]
		Culture human coronary artery smooth muscle cells			
		(HCASMC)			
Polyethylene Glycol (PEG)	Mix	3D Hydrogels	Neurites outgrowth	Biomaterials for peripheral nerve regeneration	[46]
		Culture dorsal root ganglion (DRG) cells	Control cells invasion characteristics		
Polypropylene fumarate/ tricalcium phosphate (PPF/TCP)	Mold method for PPF/TCP, scaffolds,	Porous cylinder structure	Bone growth	Biomaterials for Bone tissue engineering	[47]
	Fibrin hydrogels were pipetted into the scaffolds	Culture human gingival fibroblasts (HGFs)			
Multiwall carbon nanotube/polyurethane (MWCNT/PU)	Electrospinning for MWCNT/PU fiber, fiber fragments were incorporated into hydrogels	3D hydrogels with porous structure,	Conductivity,	Promote spinal cord regeneration	[48]
		Culture endometrial stem cells (hEnSCs)	Hydrophilicity,		
			Hydrogels stiffness,		
			Reduced degradation rate		
			Cell adhesion and proliferation		

However, a competent hydrogel for SCIs repair requires the following characteristics [48–53]:

1. the mechanical properties to anastomose spinal cord tissue;
2. the ability to combine and release peptides, proteins, and neurotrophic factors or the capacity to allow neurotrophic factors release from damaged stumps into the hydrogel;
3. the function of filling the cavity left after SCIs and reducing the scar tissue infiltration;
4. the promotion for the growth and differentiation of the implanted cells;
5. the support and guidance function for new nerve tissue or transplanted cells to migrate to the lesion site;
6. nontoxicity without incurring intense inflammations, degradability within the desired time window, and replaceability by regenerate neural tissue.

## The synthesis and modification of fibrin hydrogels

**Fig. 2:**
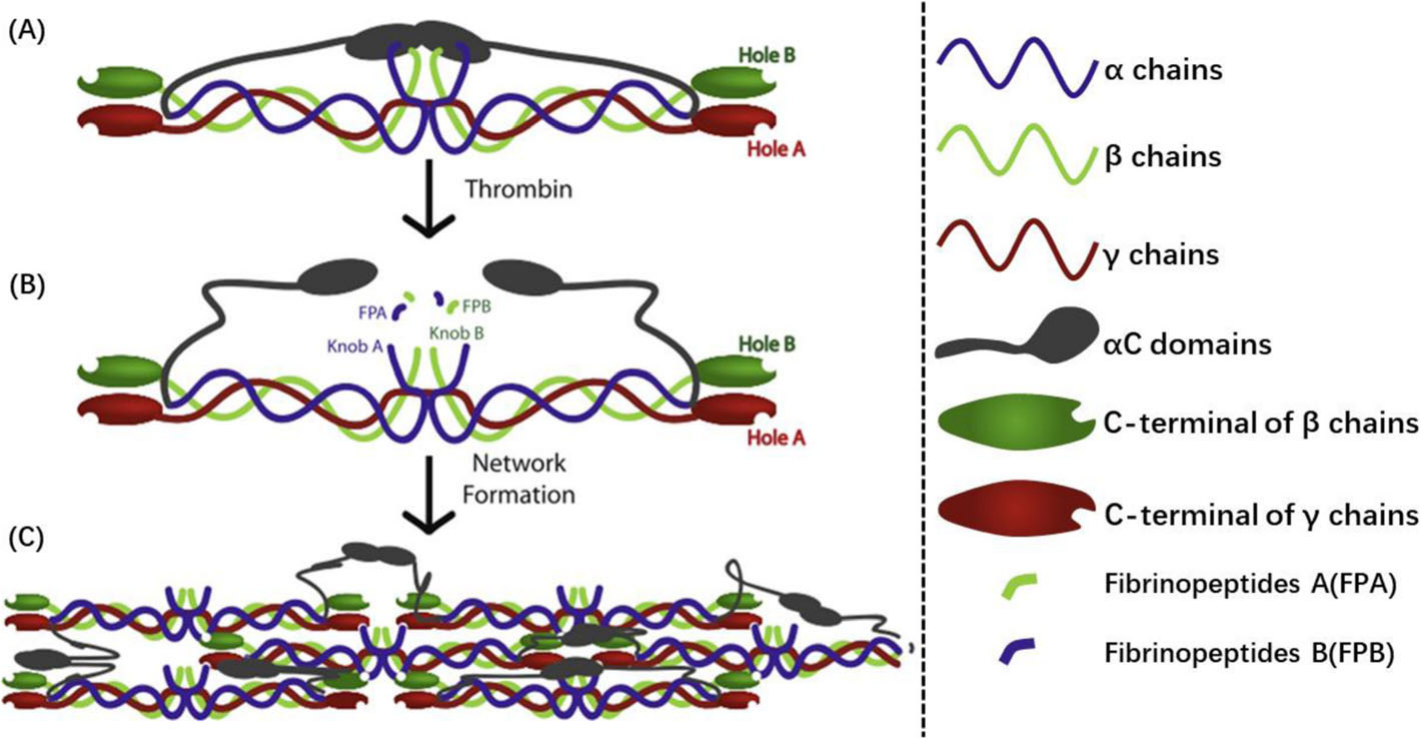
Schematic diagram of fibrin polymerization. α chains are in blue, β chains are in green, and γ chains are in red. aC domains are in gray. **a** Schematic diagram of fibrinogen monomer. **b** The catalytic action of thrombin exposes knobs A on the α chains N-terminal and knobs B on the β chains N-terminal. **c** Knobs A binds to holes a, knobs B binds to holes b, and αC domains bind to αC domains on adjacent molecules, Protofibrils finally form a branched 3D network gel structure through longitudinal extension and associate laterally. Reprinted with permission from [17]

Fibrinogen and thrombin are the primary materials to synthesize fibrin hydrogels. The simple preparation of fibrin hydrogels is the mixture of fibrinogen solution and thrombin solution [49], Fig. 2. Fibrinogen is a dimeric glycoprotein 45 nm in length and made up of three pairs of distinct chains, Aα, Bβ, and γ chains. N-terminal of six chains concatenate in the central region, and the central region connects to the end domains on both sides by a-helical coiled coils. Fibrinopeptides A (FPA) and fibrinopeptides B (FPB) lie in the central region, while the C-terminal of the α chains (known as the αC domains) is near the central region. End domains on both sides contain the C-terminal of the β and γ chains [17]. After the mixture of fibrinogen and thrombin in the required ratio, FPA and FPB on the central domain region are cleaved under the action of thrombin to expose the motif Gly-Pro-Arg (GPR) on the α-chains N-terminal (known as knobs A) and the motif Gly-His-Arg-Pro (GHRP) on the β-chains N-terminal (known as knobs B). Knob A is complementary to holes ‘a’ in the C-terminal of γ chains and knobs B is complementary to holes ‘b’ in the C-terminal of β chains. In this way, the central region of one fibrinogen molecule can combine the other adjacent fibrinogen molecule end domains. Besides, the complementary binding also takes place between αC domains of adjacent molecules [54]. Thereinto, it is believed that the A–a interaction serves as the primary driving force for the fibrin polymerization reaction, which can promote the longitudinal extension of fibrin [55]. The incomplete knowledge of the B–b interaction function reaches an agreement that closely relates to the lateral aggregation of fibrin and the stability after polymerization [56]. The mutual binding of αC domains of adjacent molecules is thought to promote the lateral aggregation of the protofibrils, and even the weak binding force does not play a decisive role [57, 58]. Protofibrils finally form a branched 3D network gel structure through longitudinal extension and associate laterally.

The ratio control of fibrinogen to thrombin can regulate the thickness of the internal fibers, the porosity of the hydrogels, and the number of branch points during the polymerization of fibrin hydrogels. Thrombin catalyzes fibrinogen to release fibrinopeptides A and fibrinopeptides B. Then, it will bind to fibrin strands and maintain catalytic activity. Therefore the binding of thrombin to new strands in the network will facilitate the new polymer formation near the network [59]. At a high thrombin concentration, the distance between ‘knob’ and ‘hole’ for complementary interaction on polymers decrease, and will produce finer fibers, smaller pores, and a higher degree of connection between fibers. Reducing the concentration of thrombin during synthesis can form a porous network and increase material modulus and ultimate tensile stress [60]. Besides, the ionic strength, PH, and other molecules present in the surrounding environment during synthesis also affect the physical the fibrin hydrogels properties. Researchers incline to the addition of molecules able to facilitate more stability and longer degradation of fibrin hydrogels, such as calcium ions, transglutaminase factor XIIIa, and anti-fibrinolytic agents including aprotinin, ε-aminocaproic acid, and α2-macroglobulin [61–68].

Apart from commercial fibrin hydrogels products [49, 69], the isolation and purification of autologous plasma can also prepare fibrin hydrogels commonly used in neural tissue engineering. However, the cost is only materials, without traffic expense and patent fee [70]. Autologous fibrin hydrogels reduce both the risk of virus transmission and concerns about non-homologous immune rejection. Besides, the cell adhesion molecules, growth factors, and cytokines in autologous fibrin can facilitate cell proliferation [71, 72]. Sharp et al. attempted to repair to hemi-cut SCI of rats by injecting hydrogel of salmon fibrin to reduce the risk of viral transmission and the occurrence rate of glial scar formation by human fibrin treatment. The result demonstrated that the rats had better motor function recovery and less abnormal pain due to the more serotonin expression at the injured end [73]. Thrombin, primarily isolated from human blood, is another critical participant in fibrin polymerization. Recently, thrombin extracted from rattlesnake venom has attracted increasing attention as an alternative to human-derived thrombin [74, 75]. Except for the similar properties to promote the repair of damage, snake venom thrombin-based fibrin hydrogels elude human-borne disease transmission due to the lack of the other human blood derivatives [76]. Spejo et al. used heterologous hydrogels synthesized by fibrinogen derived from buffalo (*Bubalus bubalis*) and thrombin-like protein derived from rattlesnake (*Crotalus durissus terrificus*) venom, which then carried mesenchymal stem cell (MSC) to repair the spinal cord ventral funiculus cut (VFC) of rats. The results discovered that the heterologous fibrin hydrogels caused up-regulation of proinflammatory cytokines at the lesion site and stimulated macrophage migration and tissue clearance; Besides, the inflammatory infiltration of the surrounding microenvironment reduced the mesenchymal stem cells’ ability to promote motor function recovery [74]. When used the same fibrin hydrogels carrying human embryonic stem cell (hESC) which can overexpress human fibroblast growth factor 2 (FGF-2) to repair ventral root avulsion (VRA) injuries of the rats, the up-regulation of anti-inflammatory factor IL-10 in the ventral corn and down-regulation of mRNA level of TNFαhas reflected the anti-inflammatory effect of hydrogels [75]. The interaction among the cells carried, the hydrogels and the damaged spinal cord, and the differences in growth, differentiation, and cytokine secretion of different types of stem cells ultimately lead to the different results in the inflammatory response of the receptors. The possible combination of maximum damage repair and minimum side effects reduction and the mechanism of interaction among stem cells, hydrogels, and damaged spinal cord are worthy of further research.

**Fig. 3:**
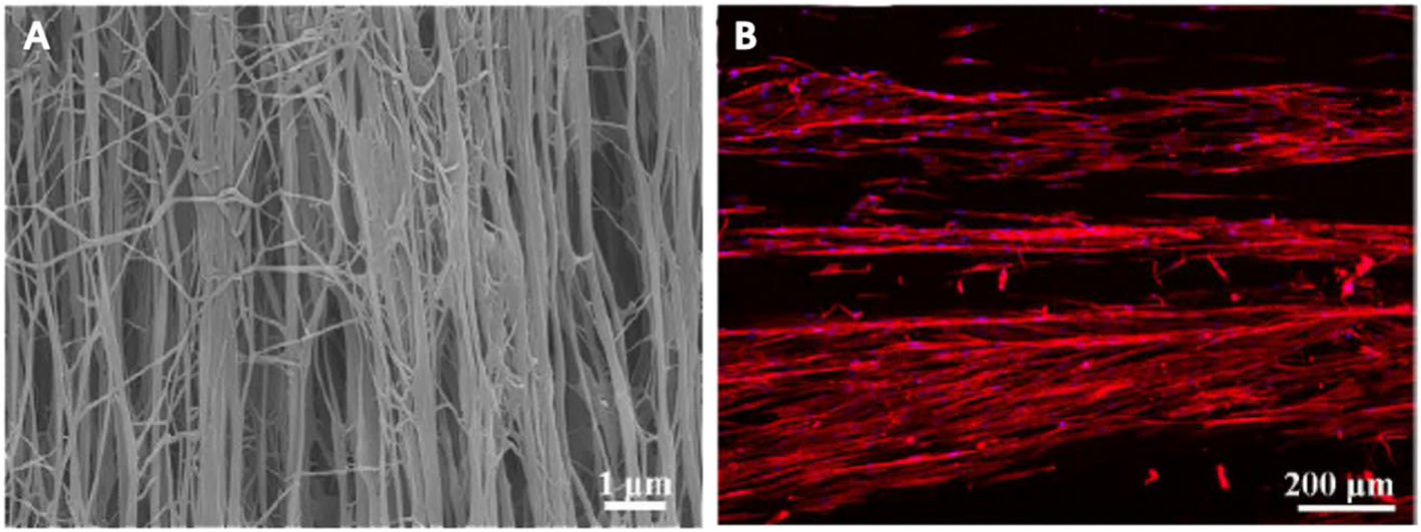
**a** Photomicrograph of electrospinning aligned fibrin hydrogel. **b** Human mesenchymal stem cells (hMCS) grow along with topographical guidance in aligned fibrin hydrogel. Adapted with permission from Zhenxia Zhang [86]

After polymerization, the fiber orientation inside the hydrogels is disordered. Compared with the non-aligned scaffolds, aligned scaffolds with a certain degree of internal guidance provide better terrain support to promote the arrangement and elongation of the regenerate axons [77–79]. Studies suggested that cells can perceive the topography changes of the adhesion area through the interaction of the cytoskeleton and ECM, and finally convert the physical signals’ adjustment into the differentiation-related genes’ expression through the transmission of focal adhesion and nuclear signals, which transform the differentiation and proliferation ability [80–83]. The fibrin hydrogels’ external shape can achieve a desired structure well fits the injury site through molding and curing during the polymerization [84]. Electrospinning [85, 86] Fig. 3, 3D printing [87], cell-mediated gel contraction [88], and other techniques can prepare the aligned fibrin hydrogel with orientation inside. Yao et al. produced fibrin hydrogels by electrospinning, which endowed the fibers with a layered arrangement. The aligned hydrogel guides the human umbilical cord mesenchymal stem cells to extend in a long spindle shape. The soft elasticity of the hydrogel (0.1–1 kPa) significantly increased the expression of neuronal differentiation markers. These two properties promote the migration and proliferation of endogenous neural cells along the fibers in the T9 hemi-cut SCI model, thereby promoting the growth of axons to the injured side [49].

**Fig. 4:**
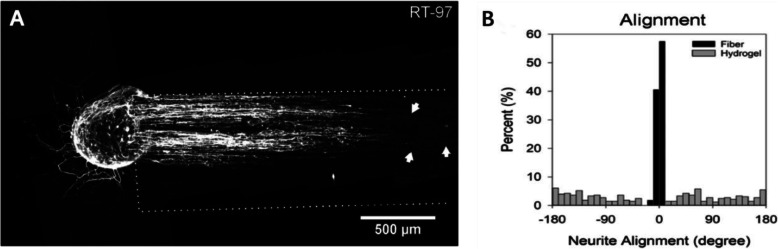
**a** Dorsal root ganglion cells were cultured in fibrin hydrogels containing 6% superparamagnetic iron oxide nanoparticles (SPIONs) and PLLA fibers. Dorsal root ganglion cells were labeled with RT-97 for neurofilament, white dotted lines Indicates the area with nerve fibers, and the white arrow indicates the three longest neurites. **b** Histograms showing the distribution of neurite growth angles (±180°), Each bar represents a 10° bin (% of total)

Electrospinning and 3D printing technology endow fibrin hydrogels with internally aligned conduits to promote damage repair. Then surgery implants internally aligned conduits fibrin hydrogels into the damaged spinal cord. This process requires the removal of part of the vertebrae, which can easily damage the surrounding intact tissue and increase the risk of infection [89, 90]. On the other hand, spinal cord tissue defect caused by compression is usually irregular. Rigid hydrogels polymerized in vitro might not anastomose the defect tissue, which reduces the hydrogels’ repairability. Injectable hydrogels can reduce unnecessary damage but lacks the internally aligned conduits [91]. Therefore, researchers developed magnetically guiding hydrogels are injectable into the spinal cord and obtain aligned fiber in situ. Based on the cascade reaction of fibrinogen and thrombin, the preparation of magnetically responsive fibrin hydrogels added substances with magnetic response capabilities, such as magnetic responsive rod-shaped microgel, magnetically responsive aligned poly-L-lactic acid (PLLA), corresponding magnetic poly (lactic-co-glycolic acid) (PLGA). An external magnetic field can guide the above magnetically responsive substances to form polydisperse particle strings, which provide the topographical guidance to the interior of the hydrogels before complete solidification [92–94] Fig. 4. Besides, the side effects of synthetic substances are noteworthy. For example, the degradation of PLGA will cause an acidic environment, generating tissue toxicity, and increasing astrocytes’ viability and inflammatory response [95–97]. The accumulation of reactive oxygen species (ROS) introduced by magnetic iron oxide nanoparticles that modify PLGA and PLLA will cause inflammation, damage to DNA, and the immune system [98].

The introduction of large molecular weight substances decreases the fibrin hydrogels’ purity, which will change the initial setting of performance or bring the adverse effects of inorganic molecules on cells. Gessmann et al. hence, mingled plasma mixtures into the synthesis chamber which has a strong electric or magnetic field to induce fibrin alignment. The viability and proliferation of the buried human mesenchymal stem cells are impervious to the electric field’s exposure during the guidance. Besides, stem cells arranged longitudinally along the direction of the aligned fibrin. During the polymerization, fibers flowing horizontally or vertically endows them with an internal directionality. The diameter of the synthetic fibrin is larger than that of the fibers synthesized in the magnetic field and electric field [99]. Berkovitch et al. employed laser photoablation to generate channels with a diameter of about 70 ± 5 μm in the hydrogel formed by ducts of synthetic polyethylene glycol and fibrinogen. The distance between the channels was 200 μm, which also guided the hydrogel. It promotes uniform tissue propagation during nerve regeneration [100].

## Fibrin hydrogels as a cell carrier

Cell transplantation has long been supposed to be one of the most probable methods for functional recovery. MSCs, neural stem cells or progenitor cells, Schwann cells, olfactory ensheathing cells, and oligodendrocyte precursor cells are in wide application to damage repair [101]. Except for filling the damaged cavity and protecting the remnant cells [102], the implanted stem cells can differentiate into motor neurons, oligodendrocytes, astrocytes, and various ventral interneurons. The regenerate neurons will connect with the upstream and downstream neurons, regulate inflammation [103], and achieve myelination in the parenchyma surrounding the injury site [104]. The brain-derived neurotrophic factor (BDNF) and the glial cell-derived neurotrophic factor (GDNF) are expressed steadily in stem cells as the nerve growth factors that promote the regeneration of the surrounding cells and blood vessels [105, 106]. Compared with the surrounding environment, the concentration gradient of growth factors also poses a guiding effect on the regeneration of autologous axons to some extent [107].

### Fibrin hydrogels carrying mesenchymal stem cells

MSCs involved in the repair of damage has proved the potential to differentiate into neurons and glial cells. The cytokines and neurotrophic factors expressed during the differentiation, the immune regulation, and the anti-apoptosis can help improve the motor function [108–110]. Compared with the plastic surface, the Arg-Gly-Asp (RGD) sequence and the larger surface endow MSCs with a higher proliferation rate and promote the differentiation to Schwann-like cells (SLCs) [111]. MSCs can function in the repair of SCIs of different types or at different phases. Mukhamedshina et al. added adipose-derived mesenchymal stem cells (ADMSCs) to wrap the rat spinal cord with 2 weeks of compression injury. The result after adding MSCs found that the down-regulation expression of the glial fibrillary acidic protein (GFAP) and the allograft inflammatory factor 1 (IBA1) suggested a decrease in astrocyte activation. Besides, the up-regulation expression of the platelet-derived growth factor β-receptor (PDGFβR) and the heat shock 70 kDa protein 1B (HSPA1b) (anti-apoptotic regulator) mRNA, prevented the secondary damage and promoted the retention of gray and white matter [102]. Compared with compression injury, spinal cord transection injury can eliminate the well-functioning nerves’ interference in therapeutic effect evaluation [112]. After the microinjection of the mixture of fibrin hydrogels and ovine bone marrow mesenchymal stem cells into the rats with spinal cord transection injury, MSCs can migrate into the spinal cord of the host and begin to differentiate into neurons and glial cells, which promote the recovery of partial motor function [113]. In the chronic phase of spinal cord transverse injury, the surgical removal of scar tissue and the addition of scar inhibitors can facilitate the fibrin hydrogel scaffolds carrying MSCs to create a microenvironment more suitable for axon regeneration, and also promote the further functional recovery of electrophysiology and motion [114]. A contrastive repair competence of various MSCs applicable for SCI transplantation suggests that adipose tissue MSCs (AD-MSCs) demonstrated the best repair effect in the motor ability recovery, inhibiting the activation of microglia and astroglia in caudal direction of injury epicenter, reducing the cavitation after trauma, and enhancing the tissue restoration. The allogeneic MSCs carried by fibrin hydrogels in the comparison conducted by implanting into SCI models are bone marrow MSCs (BM-MSCs), AD-MSCs, and dental pulp (DP-MSCs) [115].

### Fibrin hydrogels carrying neural stem/ progenitor cells

Neural stem/ progenitor cells are other cells widely used in the repair of damage. Except for the ability to reduce the formation of glial scar and reduce inflammation effectively Neural stem/ progenitor cells have a stronger neuronal differentiation tendency [116]. Likewise, fibrin hydrogels can provide embryonic stem cell-derived neural stem/progenitor cells (ES-NSPs) [117], neural progenitor cells (NPC) [118], human embryonic stem cells (hESC) [75], embryonic stem cell-derived motor neuron progenitor cells [119], and other cells, a suitable environment for proliferation and differentiation. Also, the implantation to fill the damaged site can not only reduce the cavitation after the trauma but also promote the axon extension and the motor function recovery. The flexibility of fibrin hydrogels can induce neural stem/progenitor cells (NSPC) to differentiate into dopaminergic/noradrenergic neurons and synaptic protein networks [120]. Monfared et al. acquired oligodendrocyte progenitor cells (OPC) by inducing human endometrial stem cells (hEnSCs) to overexpress miR-219, which were wrapped in fibrin hydrogels and injected into the rat spinal cord compression injury site. The results demonstrated that the OPC group achieved a higher myelination rate than the hEnSC group from where it originated. However, no significant difference appeared in the hindlimb motor function recovery between the two groups, which illustrated that myelination might not be the critical reason for motor function recovery to some extent [121].

### Fibrin hydrogels carrying other cells

Schwann cells, considered as the gold standard cell type for the treatment of peripheral injury at present, can proliferate again to longitudinally oriented bands of Bu¨ngner after damage, to support the axon orientation growth [122] and provide bioactive substances for axon migration and growth [123, 124]. Both in vitro culture and in vivo repair of sciatic nerves improved Schwann cells’ viability and supported aligned nerve tissue formation, increasing the number of neurites in the graft and distal stumps [118, 125]. However, the short survival time of the Schwann cells and the inconspicuous axon regeneration and functional recovery remain after the Schwann cells’ implantation to the spinal cord [126, 127]. Ganz et al. implanted human oral mucosal stem cells (hOMSC) into the matrix consist of fibrin, PLLA, and PLGA to repair the complete spinal cord transection injury. The implanted cells released GDNF, BDNF, and neurotrophin-3 (NT-3), in which the increased myelinated axons and neural precursors reduced the formation of glial scars [128]. The mixture of progenitor cells contained in monocytes in human peripheral blood exhibits the potential to differentiate into neural stem cells [129]. Barbizan et al. applied the fibrin hydrogels coated with monocytes through local injection to repair the ventral root-lesion of the spinal cord. The implantation increased the neurotrophic factors’ expression in the spinal cord’s ventral horn and promoted the motor function recovery [130, 131]. The same application to the dorsal root lesion also reduced glial accumulation and improved the sensory input axon plasticity [132].

### Further functional optimization on the hydrogels and the cells

The surrounding environment’ inhibition to the implanted stem cells’ proliferation and the incorrect stem cells’ proliferation will reduce the repair effect [133, 134]. Further optimization on the stem cells, the increase of the cell’s proliferation and population purity, and the culture of cell populations more propitious to nerve repair are of necessity [135]. The achievements need Further functional modification of the hydrogels and the cells.

#### Optimization on fibrin hydrogels’ function

Researchers have further processed the properties of the hydrogel matrix to achieve the requirement more suitable for cell growth. For example, fiber fragments produced by the filler of electrospun polyurethane/multi-wall carbon nanotube in the synthesis of fibrin hydrogels can improve fibrin hydrogels’ rigidity and prolong their degradation, which makes them more suitable for spinal transplantation and improve the vitality and proliferation of the cells carried [48]. The addition of perfluorotributylamine (PFTBA) during the polymerization can facilitate the remyelination and the motor function recovery. Because the high solubility for oxygen of PFTBA can release continuous oxygen, which is conducive to the cells’ survival and the growth of the microvessels in the early regeneration of the nerve [136]. Except for the application of inorganic molecules, the introduction of other proteins can also enrich the function of fibrin hydrogels. Arulmoli et al. added hyaluronic acid (HA) and laminin into the polymerization of the salmon-derived fibrin hydrogels. The result demonstrated that except for the increased rigidity and the prolonged degradation, the integrin-binding site provided by laminin could mediate the interaction between cells and hydrogel. Besides, the above fibrin hydrogels enhanced the human endothelial colony-forming cell-derived endothelial cells (hECFC-ECs)-mediated angiogenesis and supported the proliferation and differentiation of the human neural stem/progenitor cells (hNSPCs) [137]. Studies suggest that the binding of integrin to extracellular matrix ligands is essential for cells anchorage to the surrounding matrix, migration, and activation of intracellular signaling pathways. Researchers used the enzyme cross-linking of transglutaminase factor XIII to covalently bind synthetic peptide (HYD1) to fibrin hydrogels. Synthetic peptides can serve as ligands for integrin a6b1 expressed by neural stem/progenitor cells (NSPCs), Therefore, functionalized fibrin hydrogels can promote the neurite extension of cultured NSPCs and improve the motor function of SCI rats after implantation [117]. Fibrin hydrogels can not only carry the cells but also serve as an additive to the extracellular matrix hydrogel of bone or spinal cord, making the gelation rate and storage modulus of the mixed hydrogel more suitable for the transplantation into the spinal cord [138].

#### Optimization on the carried cells’ function

The filler of the various cytokines into the hydrogels carrying cells can endow the cells with the potential to differentiate more exclusively and endow the hydrogels with the potential to create an ideal microenvironment. Edgar et al. added retinoic acid and purmorphamine into the hydrogels carrying human induced pluripotent stem cells. The results exhibited that the addition promoted the carried cells’ differentiation to the spinal motor neurons, which reduced the astrocyte progenitor genes’ expression and made the neuronal population purer [139]. In the presence of basic fibroblast growth factor (bFGF), corning™ epidermal growth factor (EGF), and platelet-derived growth factor AA (PDGF-AA), fibrin hydrogels can improve cell viability and promote pluripotent stem cells’ (iPSC) differentiation into oligodendrocytes [140]. The combined application of BDNF, bFGF, cell death inhibitor (MDL28170), and vascular endothelial (VEGF) improves the transplanted cells’ survival rate, which will facilitate a wide range of stem cell-derived axonal outgrowth in the lesion [118]. In addition to the exogenous cytokines, fibrin is also a carrier for cells modified for the overexpression of basic fibroblast growth factor (FGF2). Human intervention can precisely regulate the timing of the transfected cells releasing the cytokine, which facilitates the evaluation of the efficacy of tissue cell engineering in different nerve injury phases [75].

However, some experiments suggest that fibrin carrying both cells and drugs can not always achieve superimposed advantages. Wilems et al. transplanted fibroin hydrogel-coated embryonic stem cell-derived motor neuron progenitor cells and anti-inhibitory microparticle systems (equipped with Chondroitinase ABC and nogo extracellular peptide, residues 1–40) into rat subacute SCI models. The combined application resulted in inflammation aggravation, macrophages cell infiltration, and chondroitin sulfate proteoglycans (CSPGs) increase at the transplant site, which reduced the transplanted cells’ survival rate [119]. In the experiment of Pajer et al., after implanting the human fibrin hydrogels carrying stem cells (NE-GFP-4C cell line), the disintegrated fibrin matrix induced stem cells’ death, which failed to improve the motor function and reduce the formation of glial [141]. Besides, the undesirable consequences of cell transplantation are noteworthy, such as allodynia caused by abnormal astrocyte differentiation [142], pain and spasm caused by the inappropriate functional connection of regenerative axons [143], tumors caused by excessive hyperplasia [133], abnormal activities and tissue compression caused by ectopic colonies [144], and others. Moreover, the optimal scaffold and cell combination and appropriate regulation route are worth further research.

## Fibrin hydrogels carrying therapeutic molecules and drug

Fibrin comprises many integrin peptide-binding sequences able to combine with various cells. The binding sites of fibrin to ECM protein and various growth factors enrich the means of its involvement in nerve repair [17], making fibrin hydrogels an excellent carrier candidate.

### Fibrin hydrogels carrying therapeutic molecules and drug

Fibrin hydrogels reversibly bind to various cytokines that promote the repair of SCIs, such as fibroblast growth factor (FGF), vascular endothelial growth factor (VEGF), neurotrophin-3(NT-3), and platelet-derived growth factor (PDGF) [54, 145]. The safety and feasibility of acidic fibroblast growth factor (aFGF) for the repair of chronic SCI have received extensive affirmation [146]. Fibrin hydrogels and aFGF co-injected in patients with spinal cord injury can prevent postoperative cerebrospinal fluid leakage and promote motor function recovery [147]. Hydrogels carrying fibroblast growth factor-1 can create a more permissive environment for M2 macrophages to participate in the repair of injury [148]. VEGF can be continuously released in fibrin hydrogels and promote the aggregation and differentiation of neural stem cells [149]. The T1 sequence from angiogenic inducer cellular communication network factor 1 (CCN1) covalently combined with factor XIIIa can participate in fibrin hydrogels synthesis. Furthermore, the fibrin hydrogels, after modification, illustrated a higher angiogenic function under the mediation of vascular endothelial growth factor (VEGF) [150]. The hydrogels carrying erythropoietin (EPO) can promote nerve regeneration and motor recovery in the repair of the early phase of compression injury [151]. Fibrin hydrogels also can be used as a carrier for antisense oligonucleotides (AONs), which mediates effective gene silencing by direct blocking or degrading target transcripts. Locally applied AONs effectively down-regulates the expression of glycogen synthase kinase 3 beta (GSK3β) in the injured segment, and the down-regulation of GSK3β enhances the intrinsic axon regeneration potential [152].

### Regulate the release rate of therapeutic molecules and drugs

The mixture of cytokines and drugs will face uncontrolled burst release from the polymer, which will cause high dose toxicity and drug release, reducing therapeutic effect [153]. Taylor et al. constructed a heparin-based NT-3 delivery system. Linker peptide binds to the fibrin hydrogels through covalent cross-linking and serves to sequester heparin in the fibrin hydrogels. At the same time, heparin binds NT-3 through electrostatic action, and the concentration of heparin modulates the release rate of NT-3. Fibrin hydrogels containing NT-3 increase neural fibers density in the injured spinal cord [154]. Direct addition to the fibrin hydrogels will cause the rapid degradation of chondroitinase ABC (ChABC) [155] However, after being carried by Lipid microtubes, ChABC can achieve a slow and stable release, promoting the neurites’ extension [156]. The drug delivery system for SCI’s clinical treatment has witnessed a similar means, increasing the drug targeting ability while reducing the systemic toxicity. The clinical effectiveness and stability of methylprednisolone sodium succinate (MPSS) promptly within 8 h after SCIs are crucial [157]. Polycaprolactone-based nanoparticles carrying MPSS are dispersing in fibrin hydrogels. After the implantation into acute SCI rats, the fibrin and nanoparticles protect MPSS from rapid hydrolysis, which maintains an 8 h’ therapeutic dose in the lesion site. Local application exhibited a higher targeting ability and could reduce the spinal cord injury at the level of caspase-3, which demonstrated a similar therapeutic effect to systemic high-dose methylprednisolone [158]. Immunosuppressant tacrolimus (FK506) coated with solubilized and granular partial PLGA microspheres in fibrin hydrogels can achieve a controlled release for 28 days. FK506 microspheres can facilitate more motor, and sensory neurons regenerate in rats and produce more myelinated axons at the distal end of the injury. Compared with the systemic application, no detection of FK506 is in other essential organs, except for the nerve site, avoiding systemic toxicity [159].

## Conclusions

Since its introduction, fibrin hydrogel, as one of the most promising natural polymers in neural tissue engineering, has witnessed the development and a broad application in neural tissue engineering, bone tissue engineering, skin tissue engineering, and other fields. Despite the advantages mentioned above, fibrin-based hydrogels still face many challenges. Despite the advantages of biocompatibility, modifiability, and the ability to carry multiple cells and therapeutic factors, challenges continue in the clinical application of fibrin-based hydrogels. The FDA has approved many materials for peripheral nerve injuries repair [160]. A functional scaffold shall meet the clinical requirements of biocompatibility and interaction with cell tissue except for access to the specific physical, chemical, mechanical, and degradation properties. Besides, ethics, government regulation, development cost, clinician preference, and others are the issues involved [161]. Researchers tend to evaluate the repair effect by implanting hydrogel scaffold into animal models of spinal cord transection or hemisection. The animal models’ tissue defects are regular, different from the irregular ones of human traumatic SCIs, usually with vertebrae, intervertebral discs, and ligament injuries. Irregular tissue defects may generate inaccurate implantation of scaffold and inconsonant therapeutic effects on different levels of damage [162]. Injectable fibrin hydrogel can polymerize in vivo to anastomose the irregular defects but fails to provide topographical guidance to cells. Magnetic response injectable hydrogels can solve the preceding problem by magnetically positioning in situ, and providing consistent topographical guidance. The use of heterologous hydrogels can reduce the risk of blood-borne diseases, but the retention of heterologous substances in the body can also trigger a specific inflammatory response. When fibrin hydrogels carry cells, cytokines, or drugs in the repair of SCIs, the therapeutic effect depends on the function of the carried substances. The structure and intrinsic potential of hydrogel still need further modification and improvement, the interaction of hydrogels with cells and therapeutic agents warrants further investigation. Briefly, the support of increasing cross-linking and modification technologies will unlock more potentials of fibrin hydrogels in neural tissue engineering.

## Data Availability

Not applicable.

## Abbreviations

SCIs: Spinal cord injuries
ECM: Extracellular matrix
CNS: Central nervous system
FDA: Food and Drug Administration
CSPG: Chondroitin sulfate protein
MAIs: Myelin-associated inhibitors
FPA: Fibrinopeptides A
FPB: Fibrinopeptides B
MSC: Mesenchymal stem cell
hESC: Human embryonic stem cell
FGF: Human fibroblast growth factor
PLLA: Poly-L-lactic acid
PLGA: Poly (lactic-co-glycolic acid)
BDNF: Brain-derived neurotrophic factor
GDNF: Glial cell-derived neurotrophic factor
ADMSCs: Adipose-derived mesenchymal stem cells
NSPC: Neural stem/progenitor cell
OPC: Oligodendrocyte progenitor cells
hENSCs: Human endometrial stem cells
NT-3: Neurotrophin-3
PFTBA: Perfluorotributylamine
VEGF: Vascular endothelial
ChABC: Chondroitinase ABC
MPSS: Methylprednisolone sodium succinate
